# Early prediction of blood stream infection in a prospectively collected cohort

**DOI:** 10.1186/s12879-021-05990-3

**Published:** 2021-04-02

**Authors:** David Nestor, Hanna Andersson, Pernilla Kihlberg, Sara Olson, Ingrid Ziegler, Gunlög Rasmussen, Jan Källman, Sara Cajander, Paula Mölling, Martin Sundqvist

**Affiliations:** 1 Department of Laboratory Medicine, Clinical Microbiology, Faculty of Medicine and Health, Örebro University, Örebro, Sweden; 2 Department of Infectious Diseases, Örebro University Hospital, Örebro, Sweden; 3 School of Medical Sciences, Faculty of Medicine and Health, Örebro University, Örebro, Sweden

**Keywords:** Bacteremia, Sepsis, Clinical decision rules

## Abstract

**Background:**

Blood stream infection (BSI) and sepsis are serious clinical conditions and identification of the disease-causing pathogen is important for patient management. The RISE (Rapid Identification of SEpsis) study was carried out to collect a cohort allowing high-quality studies on different aspects of BSI and sepsis. The aim of this study was to identify patients at high risk for BSI who might benefit most from new, faster, etiological testing using neutrophil to lymphocyte count ratio (NLCR) and Shapiro score.

**Methods:**

Adult patients (≥ 18 years) presenting at the emergency department (ED) with suspected BSI were prospectively included between 2014 and 2016 at Örebro University Hospital. Besides extra blood sampling, all study patients were treated according to ED routines. Electronic patient charts were retrospectively reviewed. A modified Shapiro score (MSS) and NLCR were extracted and compiled. Continuous score variables were analysed with area under receiver operator characteristics curves (AUC) to evaluate the ability of BSI prediction.

**Results:**

The final cohort consisted of 484 patients where 84 (17%) had positive blood culture judged clinically significant. At optimal cut-offs, MSS (≥3 points) and NLCR (> 12) showed equal ability to predict BSI in the whole cohort (AUC 0.71/0.74; sensitivity 69%/67%; specificity 64%/68% respectively) and in a subgroup of 155 patients fulfilling Sepsis-3 criteria (AUC 0.71/0.66; sensitivity 81%/65%; specificity 46%/57% respectively). In BSI cases only predicted by NLCR> 12 the abundance of Gram-negative to Gram-positive pathogens (*n* = 13 to *n* = 4) differed significantly from those only predicted by MSS ≥3 p (*n* = 7 to *n* = 12 respectively) (*p* < 0.05).

**Conclusions:**

MSS and NLCR predicted BSI in the RISE cohort with similar cut-offs as shown in previous studies. Combining the MSS and NLCR did not increase the predictive performance. Differences in BSI prediction between MSS and NLCR regarding etiology need further evaluation.

**Supplementary Information:**

The online version contains supplementary material available at 10.1186/s12879-021-05990-3.

## Introduction

Blood stream infection (BSI) is a serious condition associated with high morbidity and mortality [1] with an annual incidence of 0.04–0.1% in community-acquired infections [2]. There is a risk for BSI patients to develop sepsis, a “life-threatening organ dysfunction caused by a dysregulated host [immune] response” [3]. Although sepsis may occur despite no bacterial invasion into the blood stream, microorganisms isolated from blood are often considered as the causative agent of the sepsis episode and are used to tailor the antibiotic treatment. Patients with BSI requires prompt medical attention, and for those at risk of developing septic shock, timely antibiotic therapy is crucial since mortality may be increased if initiation is delayed [4]. This is contrasted by the 24–72 h it usually takes to identify the disease-causing pathogen using blood culture (BC) [5]. Faster methods using molecular techniques (e.g. multiplexed PCR and metagenomic approaches) for pathogen identification [6, 7] are promising and will probably play a part in future routine diagnostics [8].

It would be valuable to identify patients with a high risk of BSI, which could benefit the most from novel, fast, pathogen identification methods, and several BSI prediction tools have been proposed. These have been single biomarkers (e.g. C-reactive protein [9], serum procalcitonin [10] and serum lactate [11]) or a combination of clinical parameters and biomarkers [12–14]. The Shapiro score [15], originally developed to rule out patients with low risk of positive blood culture, is one of those validated tools [16–18] and the differential count ratio of neutrophils and lymphocytes (NLCR), reflecting physiological stress response [19], has in addition recently been shown to predict BSI [20–22].

During 2014, the RISE-study (Rapid Identification of SEpsis) was conducted at the Department of Infectious Diseases, Örebro University Hospital. The objective was to establish a well-defined cohort of patients with suspected BSI/sepsis to utilize in the development and evaluation of rapid methods to identify pathogens and biomarkers indicative of BSI and sepsis*.* The aim *of the present study was to describe the RISE-cohort and to assess if NLCR and/or the Shapiro score could be used to predict BSI in this cohort.*

## Patients and methods

### Study design, setting and inclusion criteria

The study was performed at the Örebro University Hospital with about 550 beds, 1000 daily visits to the hospital’s clinics, and a catchment area of around 290,000 people [23]. The RISE cohort was included prospectively by consecutive enrollment of adult patients (≥18 years) who presented day-time and evenings to the infectious disease emergency department (ED) and were subjected to blood culture due to suspected BSI. No exclusion criteria were predefined other than disapproval of study participation. With an estimated rate of positive blood cultures at 12% about 800 patients were planned to be included to reach 100 cases with positive blood culture. Enrollment in the study did not change the clinical assessment in any aspect.

### Laboratory investigations

Blood culture (recommended routine: two sets of aerobic and anaerobic bottles) was obtained either through a single venipuncture or through two independent samplings using BACTEC™ vials (BD, Sparks, MD US). Blood cultures were transferred to the Department of Laboratory Medicine, Clinical Microbiology, University Hospital, Örebro and incubated in semi-automated BC-cabinets (BACTEC™, BD, Franklin Lakes, NJ US) until signaling positive or for 7 days until reported negative. In cases with suspicion of endocarditis the incubation time was extended to 14 days. Blood cultures positive with coagulase-negative staphylococci spp. and *Cutibacterium* spp. were considered positive only if growth in at least two BC bottles and the culture result had prompted adjusted antibiotic treatment to the isolated pathogen, but else regarded as contaminants. On all included patients additional sampling was performed consisting of one extra aerobic BC bottle (BD, Sparks, MD US), 2 × 10 ml of whole blood in EDTA tubes, 3 ml of whole blood in RNA stabilizing tubes and 5 ml whole blood in serum tubes. The samples were sent to the laboratory with the same time delay as for clinical samples. When arriving at the laboratory all samples were treated according to the study protocol and stored frozen (− 70 °C).

### Clinical data

Scanned ED-charts and electronic medical records were retrospectively reviewed by one of four physicians (DN, HA, PK, SO). The following data were extracted: demographics (gender, age); medical history (comorbidities as defined by Charlson et al. [24]), previous medication (antibiotic treatment within 14 days prior to or at inclusion, immunosuppressive medication); vital signs at presentation and during the stay at ED; clinical chemistry status at arrival, history of present illness (duration, symptoms); presumptive diagnose; initial assessment (admission for in-patient care, commenced antibiotic treatment if any); duration of in-patient care and continuation of antibiotic treatment (treatment changes, antibiotic agent, administration route (oral/intravenous)); primary diagnose at discharge and 28 and 90 days mortality. A comprehensive list of all extracted variables is available in Supplementary material 1.

### Assessment of clinical scores and other predictors of BSI

Shapiro score was, in line with previous studies [17], assessed using a modification of the original definition [15] as all variables but band cells (not routinely measured at the study place) were extracted and compiled (Table 1), hereafter referred to as Modified Shapiro score (MSS). Organ dysfunction caused by sepsis was defined according to the latest Sepsis-3 definitions [3]. Baseline SOFA score was evaluated from medical history and laboratory examinations from before inclusion. Glasgow Coma Scale score was converted from the Swedish Reaction level scale (RLS) [25]. NLCR was computed from the white blood cell differential count.

**Table 1:** The original Shapiro score and decision rule to identify patients in need of blood culture testing. Modified from [15]

Major criteria	Minor criteria (1 point)
Suspected endocarditis (2 points)	Age > 65 years
Temperature > 39.4 °C (103.0 °F) (3 points)	Temperature 38.3–39.3 °C
Indwelling vascular catheter (3 points)	Chills
	Hypotension (Systolic blood pressure < 90 mmHg)
	White blood cell count > 18,000 cells/mm3
	Platelets < 150,000 cells/mm3
	Creatinine > 2 mg/dL
	Band percentage > 5%^a^

One major criterion or two or more minor criteria advise blood culture testing

^a^Not available in this study

### Statistical analysis

Clinical data are presented as percent of the total population and the respective blood culture status groups (positive or negative/contamination). The difference in distribution was calculated using Pearson chi-square or Fisher exact test when appropriate. Continuous variables are presented with median/interquartile range (IQR) (non-parametric variables) and mean/standard deviation (SD) (parametric variables). Differences were calculated using Mann Whitney and students t-test when suitable. Area under the receiver operator characteristics curves (AUC) were performed to measure the predictive capacity of continuous variables. Differences in AUC values were analysed using the method for dependent receiver operator characteristics curves by DeLong et al. [26]. Binary logistic regression was used to measure the combined predictivity of two parameters and the Youden index indicated the optimal cut-off for sensitivity and specificity for each parameter. The sensitivity and specificity of different parameters were compared pairwise using the McNemar test by analysing the blood culture positive and negative (including false positive/contaminated) separately [27]. MedCalc Software (v 19.1.3) was used for all statistical analyses. Differences were considered statistically significant at *p* < 0.05. All methods were carried out in accordance with relevant guidelines and regulations.

## Results

### Patient characteristics

From October 2014 to October 2016, 517 events of suspected BSI were initially sampled in the RISE-study (patients *n* = 505). Thirty-three (6%) did not fulfill inclusion criteria (i.e. no written consent within 14 days) and were therefore excluded, leaving 484 events (patients = 474) in the final cohort, hereafter referred to as patients. The majority of patients (68%, *n* = 328) were included during the first 12 months. Patient characteristics are summarized in Table 2.

**Table 2:** Baseline characteristics overall and separated by blood culture positivity and negative or contaminated blood cultures

Characteristics	Total ***n*** = 484	Missing value n (%)	Blood culture		
			Positive ***n*** = 84	Negative/contamination ***n*** = 400	***p***
**General characteristics**					
Age, mean (SD)	64.6 (±19.3)	0	69.6 (±17.0)	63.6 (±19.6)	0.03
Male sex	272 (56.1)	0	52 (61.9)	220 (54.9)	ns
**Comorbidities**					
Diabetes	69 (14.2)	0	12 (14.3)	57 (14.2)	ns
Heart failure	64 (13.2)	0	14 (16.7)	50 (12.5)	ns
Chronic obstructive pulmonary disease	39 (8.0)	0	8 (9.5)	31 (7.7)	ns
Kidney failure	20 (4.1)	0	5 (6.0)	15 (3.7)	ns
**Charlson comorbidity index**					ns
- 0	211 (43.5)	0	33 (39.3)	178 (44.4)	
- 1	99 (20.4)	0	17 (20.2)	82 (20.4)	
- > 2	175 (36.1)	0	34 (40.5)	141 (35.2)	
**Clinical scores**					
SIRS criteria fulfilled	325 (67.0)	6 (1.2)	70 (83.3)	255 (63.6)	< 0.05
qSOFA ≥2 points	246 (50.7)	0	47 (56.0)	199 (49.6)	ns
SOFA score, median (IQR)	1 (0–2)	0^a^	1 (0–2)	1 (0–2)	ns
Sepsis-3 Criteria fulfilled	155 (32)	0^a^	32 (38)	123 (30.8)	ns
Modified Early Warning Scale, Median (IQR)	3 (2–5)	18 (3.7)	4 (2–5)	3 (2–5)	< 0.05
Shapiro ≥2 points	320 (66.1)	0	72 (85.7)	248 (61.8)	< 0.001
Shapiro score, median (IQR)	2 (1–3)	0	3 (2–4)	2 (1–3)	< 0.001
**Antibiotic therapy**					
Antibiotic treatment at inclusion	89 (18.4)	0	4 (4.8)	85 (21.2)	< 0.001
Antibiotic prescription < 14 before inclusion	85 (17.6)		10 (11.9)	75 (18.8)	ns
**Suspected infection site**					
Lower respiratory tract	163 (33.6)	0	25 (29.8)	138 (34.4)	ns
Urinary tract	120 (24.7)	0	42 (50.0)	78 (19.5)	< 0.001
Skin and soft tissue	88 (18.1)	0	13 (15.5)	75 (18.7)	ns
Abscess	33 (6.8)	0	4 (4.8)	29 (7.2)	ns
**Vital parameters at admission. Median (IQR)**					
Respiratory rate	21 (18–26)	13(2.7)	23 (18–30)	20 (18–25)	ns
Pulse rate, beats per minute	94 (81–106)	4 (0.8)	100 (77–110)	93 (82–106)	ns
Body temperature °C	38.4 (37.7–39.1)	7 (1.4)	38.8 (38.1–39.6)	38.3 (37.6–39.0)	< 0.001
Systolic blood pressure, mm Hg	135 (120–150)	4 (0.8)	134 (120–155)	135 (120–150)	ns
Diastolic blood pressure, mm Hg	79 (70–85)	13 (2.7)	75 (61–85)	80 (70–85)	ns
Mean Arterial Pressure, mm Hg	96 (87–106)	13 (2.7)	93 (83–107)	97 (87–105)	ns
**Laboratory values. Median (IQR)**					
WBC × 10^9^/L	11.5 (8.7–15.2)	8 (1.6)	13.7 (9.2–17.1)	11.2 (8.6–14.5)	< 0.05
Neutrophil cell count × 10^9^/L	9.2 (6.3–12.5)	12 (2.5)	11.3 (7.2–14.8)	8.7 (6.2–11.9)	< 0.05
Neutrophil Lymphocyte count ratio (NLCR)	9.3 (5.1–16.1)	16 (3.3)	16.2 (10.0–25.1)	8.2 (4.7–14.6)	< 0.001
Serum lactate (mmol/L)	1.6 (1.2–2.2)	53 (10.9)	2.1 (1.4–2.8)	1.6 (1.2–2.0)	< 0.001
Lymphocyte cell count × 10^9^/L	1.0 (0.7–1.5)	16 (3.3)	0.7 (0.3–1.2)	1.1 (0.7–1.6)	< 0.001
Creatinine (μmol/L)	84.0 (68–107)	14 (2.9)	91 (73–128)	83 (67–106)	< 0.05
Bilirubine (μmol/L)	13.0 (10–18)	24 (4.9)	15 (12–19)	13 (9–18)	< 0.05
C-reactive protein (mg/L)	98 (41–198)	1 (0.2)	109 (47–196)	98 (40–198)	ns

^a^SOFA-score and Sepsis-3 was calculated in all patients although some patients were missing individual values for SOFA-criteria

### Microbiological investigations

Four hundred sixty-five (96%) of the blood cultures consisted of at least four culture bottles. Of the 484 included patients, 99 (20%) had positive blood cultures, and 84 (85%) of these were considered clinically relevant with a total of 90 bacterial isolates (Table 3). The most common clinically relevant isolated pathogens were *Escherichia coli* (*n* = 32) and *Staphylococcus aureus* (*n* = 9) (Table 3).

**Table 3:** Bacteria isolated (*n* = 90) from 84 positive blood cultures deemed clinically relevant (light gray columns) and the number of these blood cultures within three groups: Patients who fulfilled Sepsis-3 criteria (155 patients), patients with a Neutrophil to lymphocyte count ratio (NLCR) > 12 (177 patients) and patients with a Modified Shapiro score (MSS) ≥3 points (195 patients). The “Total” represents the number (percentage) of Gram-negative/−positive findings in each group

Gram-negative isolates	n	Sepsis-3 (*n* = 155)	NLCR > 12 (*n* = 177)	MSS ≥ 3p (*n* = 195)	Gram-positive isolates	n	Sepsis-3 (*n* = 155)	NLCR > 12 (*n* = 177)	MSS ≥ 3p (*n* = 195)
*Escherichia coli*	32	12	25	21	*Staphylococcus aureus*	9	4	6	7
*Klebsiella pneumoniae*	5	1	4	3	Alfa-hemolytic streptococci	9	3	4	2
*Bacteroides fragilis*	3	1	1	2	*Streptococcus pneumoniae*	5	2	2	3
*Pseudomonas aeruginosa*	2	1	1	2	Beta-hemolytic streptococci (Non-group A)	5	2	5	4
*Salmonella panama*	1				*Enterococcus faecalis*	2	1	1	2
*Neisseria meningitidis*	1	1	1	1	Coagulase Negative Staphylococci	3	3	2	2
*Francisella tularensis*	1			1	*Aerococcus urinae*	1	1		1
*Fusobacterium necrophorum*	1	1	1		*Listeria monocytogenes*	1			1
*Klebsiella oxytoca*	1			1	*Cutibacterium acnes*	1		1	1
*Enterobacter cloacae*	1	1	1	1	*Parvimonas micra*	1			
*Pasteurella multocida*	1		1	1	*Peptostreptococcus anaerobius*	1		1	
*Citrobacter* sp.	1		1		*Clostridium perfringens*	1		1	1
*Pseudomonas* sp. (Non-aeruginosa)	1	1	1	1					
Total	51	19 (37%)	37 (73%)	33 (65%)		39	16 (41%)	23 (59%)	27 (69%)

### Laboratory parameters and scoring systems to predict BSI

**Fig. 1:**
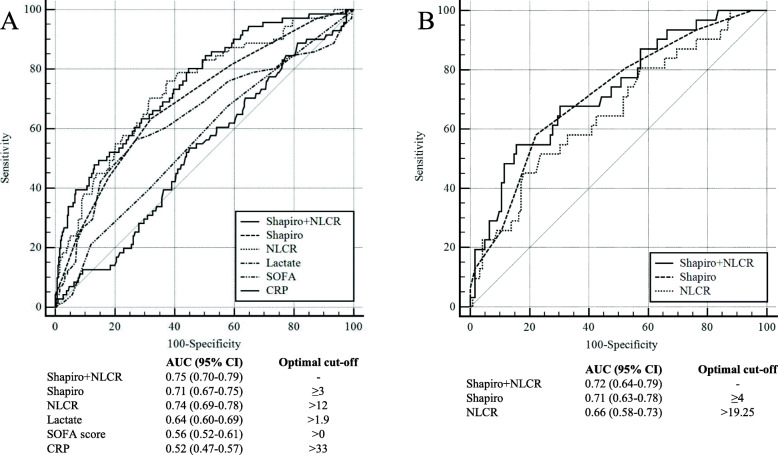
Receiver operating characteristic (ROC) curves for parameters differentiating BSI from non-BSI in (**a**) all patients (*n* = 484) and (**b**) patients fulfilling sepsis-3 criteria (*n* = 155). The AUC value for NLCR alone were significantly higher than Lactate, SOFA score and CRP (**a**) (*p* < 0.05). The AUC value for Shapiro+NLCR was significantly higher than all other but NLCR (*p* < 0.05) (**a**). Due to the limited predictive performance of Lactate, SOFA score and CRP only Shapiro score and/or NLCR was used in (**b**). In (**b**) no statistical significant difference was observed between the individual AUC values

As the decision on the analysis of clinical chemistry was not guided in the study not all patients were analysed for all markers (Table 2). NLCR was available in 468 patients (97%). Neutrophil cell count, NLCR, serum lactate and MSS differed significantly between patients with positive and negative blood culture status (Table 2). All AUC values for differentiating BSI from non-BSI are presented in Fig. 1a and all AUC values except for CRP reached statistical significance. The recommended cut-offs for MSS and NLCR were ≥ 3 points and > 12, respectively*.* Sensitivity, specificity, predictive values and likelihood ratio calculations for MSS and NLCR at different cut-offs are presented in Table 4.

**Table 4:** Sensitivity, specificity, positive (PPV) and negative (NPV) predictive values and positive (LR+) and negative (LR-) likelihood ratios for predicting positive blood culture using cut-off values of Neutrophil-lymphocyte count ratio (NLCR) and modified Shapiro score (MSS) suggested previously and based on the data presented in this study. Values presented in the whole cohort and among patients fulfilling sepsis-3 criteria. 95% confidence interval presented within brackets. Data for patients not fulfilling sepsis-3 criteria are presented in Supplementary Table 3

	Whole cohort *n* = 484 (positive blood cultures *n* = 84 (17%))						Patients fulfilling Sepsis-3 criteria *n* = 155 (positive blood cultures *n* = 32 (21%))					
	Sensitivity (%)	Specificity (%)	PPV (%)	NPV (%)	LR+	LR-	Sensitivity (%)	Specificity (%)	PPV (%)	NPV (%)	LR+	LR-
NLCR > 10	74 (64–83)	60 (55–65)	28 (25–32)	92 (88–94)	1.9 (1.6–2.2)	0.4 (0.3–0.6)	77 (59–90)	43 (35–53)	26 (21–31)	88 (79–94)	1.4 (1.1–1.7)	0.5 (0.3–1.0)
NLCR > 12	67 (56–77)	68 (64–73)	31 (27–36)	91 (88–93)	2.1 (1.7–2.6)	0.5 (0.4–0.7)	65 (45–81)	57 (48–66)	28 (22–35)	86 (79–91)	1.5 (1.1–2.1)	0.6 (0.4–1.0)
MSS ≥ 2	86 (76–92)	38 (33–43)	23 (21–25)	93 (88–96)	1.4 (1.2–1.6)	0.4 (0.2–0.6)	94 (79–99)	24 (17–33)	24 (22–27)	94 (79–98)	1.2 (1.1–1.4)	0.3 (0.1–1.0)
MSS ≥ 3	69 (58–78)	64 (59–69)	29 (25–33)	91 (88–93)	1.9 (1.6–2.3)	0.5 (0.3–0.7)	81 (64–93)	46 (37–56)	28 (24–33)	91 (82–95)	1.5 (1.2–1.9)	0.4 (0.2–0.9)
NLCR> 12 and MSS ≥ 3	48 (36–59)	84 (80–88)	39 (32–47)	88 (86–90)	3.0 (2.2–4.2)	0.6 (0.5–0.8)	52 (33–70)	73 (64–81)	33 (24–43)	86 (80–90)	1.9 (1.2–3.0)	0.7 (0.5–1.0)

A complete SOFA score was available in 446 (92%) of the patients, yet SOFA score was calculated on available data in all patients. In the subgroup of 155 patients fulfilling Sepsis-3 criteria, 32 patients (21%) were blood culture positive compared to 52 of the 329 patients in the non-sepsis group (16%) (ns). The ability of MSS and/or NLCR in differentiating BSI from non-BSI in the sepsis group is presented in Fig. 1b. Corresponding optimal cut-off values were ≥ 4 points (MSS) and ≥ 19.25 (NLCR) (Fig. 1b). Sensitivity, specificity, predictive values and likelihood ratio calculations for these cut-offs are found in Supplementary material 2 and 3.

### Diagnostic agreement of MSS and NLCR

At optimal cut-offs in this cohort, the MSS (≥3 p) and NLCR (> 12) had equal sensitivities (69, 67%) (*p* = 0.86) and specificities (64, 68%) (*p* = 0.17) to predict BSI. The groups of patients with either MSS ≥ 3 p *or* NLCR > 12 were equally large (196/484, 40% and 177/468, 38% respectively) but only 100 patients (21%) had both MSS ≥ 3 p *and* NLCR > 12. Thirty-nine of these 100 patients (39%) had positive blood culture. Among patients fulfilling Sepsis-3 criteria (*n* = 155), MSS ≥3 p and NLCR > 12 predicted BSI with similar sensitivities (81, 65%) (*p* = 0.27) and specificities (46, 57%) (*p* = 0.07). Among patients fulfilling Sepsis-3 the groups with either MSS ≥3 p (87/155, 56%) *or* NLCR > 12 (72/153, 47%) were equally large and 48 (31%) had both. Of these 48 patients, 16 (33%) were blood culture positive.

**Fig. 2:**
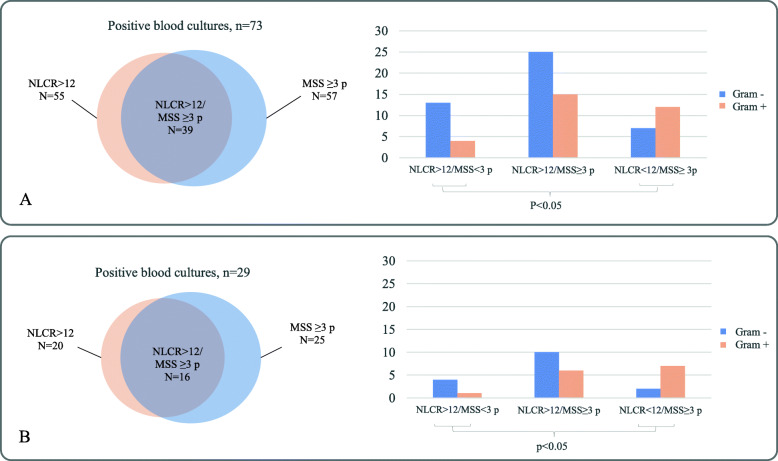
Venn diagrams on the left represent the number of true positive blood cultures predicted by either neutrophil to lymphocyte count ratio (NLCR) (orange) and/or Modified Shapiro score (MSS) (blue) in (**a**) the whole cohort, *n* = 73, and (**b**) among patients fulfilling Sepsis-3 criteria, *n* = 29. Bar charts represent the corresponding Gram stain results (Gram-negative blue and Gram-positive orange) in all isolated pathogens in from (**a**), *n* = 76 and (**b**), *n* = 30. Significantly skewed distribution of Gram negative vs Gram positive culture results are marked with staples

In total, 73 of 84 (87%) positive blood cultures were predicted by either MSS ≥3 p or NLCR > 12. The corresponding number among patients fulfilling Sepsis-3 was 29 of 32 (91%), see Fig. 2. In BSI cases only predicted by NLCR> 12 the abundance of Gram-negative to Gram-positive isolated pathogens (*n* = 13 to *n* = 4) differed significantly from those only predicted by MSS ≥3 p (*n* = 7 to *n* = 12 respectively) (*p* < 0.05), Fig. 2a. This skewed distribution was also seen among patients fulfilling Sepsis-3 criteria (Fig. 2b).

## Discussion

The diagnosis of BSI and sepsis is challenging both for the clinician and the laboratory as no clinical score, biomarker or microbiological test shows optimal predictive value and/or speed [9, 14, 21, 28]. In this study NLCR and Shapiro score showed equal performance in predicting positive blood culture; both in the entire cohort of patients as well as in the subgroup of patients fulfilling Sepsis-3 criteria.

At presentation in the ED, scores based on clinical parameters and/or bedside biomarkers are the most rapid way of classifying the risk of BSI and/or sepsis can be used to guide both diagnostic activities and initial treatment decisions. The use of NLCR in the prediction of positive blood culture have been studied previously with reported AUC values of 0.68–0.77 [20–22, 29] consistent with our results (0.74 (0.69–0.78)). No consensus regarding use of cut-off level has been reached. Arbitrarily, a cut-off at 10 has most commonly been used and a cut-off at 12 (supported by this study) has also been discussed [14, 20–22]. In this study, a relatively high prevalence of positive blood cultures (and theoretically over-all higher NLCR values) could have contributed to the higher cut-off observed. The cohort displayed BC positivity in 84 of 484 (17.4%) which is higher than the 12.5% (197/1572) reported in a similar study consisting only of patients with suspected sepsis [14] but lower than 26.9% (147/558) in another recent cohort study consisting of patients with more severe sepsis and abnormal vital signs [30]. The high prevalence probably mirrors that the patients were included through the ED at the Infectious Disease Department at the hospital and thus with a higher probability of BSI. The inclusion rate decreased throughout the study period, however the percentage of positive cultures, median Charlson, and SOFA remained stable (data not shown).

The recommended cut-off for Shapiro score is ≥2 points [15]. However, the Shapiro score was originally developed to avoid unnecessary ordering of blood cultures and has a low cut-off with focus on high sensitivity. The cut-off at ≥3 points in this study decreased the sensitivity of BSI prediction from 86 to 69% but raised the specificity from 38 to 64% compared to a cut-off at ≥2 points and was therefore used. The AUC value of 0.71 was lower than reported in earlier studies (0.75–0.83) [15–18], probably due to the exclusion of the band cells from the original score. Combining the MSS with NLCR (as indicator of neutrophil cell response) increased the AUC value to 0.75 compared to MSS alone. The prevalence of positive blood cultures among patients with MSS ≥3 (*n* = 57, 29%) in this study was markedly higher than 8–10% reported in similar studies [15, 18], probably reflecting the overall high BC prevalence discussed above. Among patients fulfilling Sepsis-3 higher cut-off values were calculated for both MSS (≥4 points) and NLCR (> 19.25) for optimal balance between sensitivity and specificity. However, the relative complexity of using different cut-offs depending on Sepsis-3 status (requiring prior calculation of SOFA-score) warrants the use of the same cut-off value (Shapiro≥3 or NLCR> 12) irrespective of sepsis status in an ED setting. CRP and serum lactate was considered not suitable as predictors of BSI due to significantly lower AUC values, consistent with earlier studies [9, 11, 29], and were therefore not further evaluated. Elevated levels of CRP and lactate are likely too nonspecific indicators of inflammation and hypoxia/critical illness to be useful in the prediction of positive blood culture. Elevated lactate levels at admission may instead be useful in predicting sepsis outcome [31]. Unfortunately, procalcitonin levels were only analysed in a few patients at admission and could thus not be evaluated.

Interestingly, NLCR alone correctly predicted a higher number of Gram-negative pathogens than MSS and conversely, the MSS alone predicted a higher number of Gram-positive pathogens (Table 3). These differences were consistent even in the subgroup of patients fulfilling Sepsis-3 criteria (Fig. 2). This is contrasted by the result from Laukemann et al. where Shapiro score resulted in higher AUC values for Gram-negative pathogens [18]. The presence of band cells, or immature neutrophils, were excluded from the original Shapiro score (Table 1) and might explain the reduced prediction of Gram-negative BSI as it indicates early neutrophil response [32]. Nine patients with MSS < 3/NLCR> 12 and positive blood culture had two Shapiro score points and were at risk to be “false negative” due to exclusion of the band cells criteria (Table 1). NLCR also failed to predict some of the Gram-positive cultures. In a retrospective study by Turak et al., 121 patients with infective endocarditis (mostly caused by Gram-positive pathogens) had low NLCR at admission (7.3 ± 3.0) [33] making an NLCR cut-off at 12 less suitable in these cases. In a recent study by Marik et al. Gram-negative BSI rendered higher NLCR values compared to Gram positive BSI (17.3 vs 12.5) [29]. This together with the results presented here indicates that NLCR predicts Gram-negative BSI better than Gram-positive BSI but this still needs to be evaluated in larger materials. Regarding diagnostic agreement between NLCR and MSS the populations predicted to have positive blood culture were found to be rather separated despite similar AUC values. Overall, NLCR and MSS showed complementary rather than additive predictive capacity in the cohort studied here.

As the RISE-study was launched in 2014, the clinical evaluations were not optimized for the Sepsis-3 criteria which were released in 2016 [4]. This forced a retrospective assessment of SOFA-score and one or more SOFA variables were missing in 38 patients (8%) whereas SIRS criteria (Sepsis-2) were available in nearly all patients (*n* = 6, 1% missing values). Despite this limitation, Sepsis-3 criteria were used as it has repeatedly been shown to identify sepsis patients at higher risk for mortality and poor outcomes compared to Sepsis-2 [34]. No exclusion criteria (accept age < 18 years) were used and patients with pre-existing medical conditions potentially impairing the immune response (e.g. long-term steroid treatment, hematologic malignancies) and affecting NLCR and/or Shapiro score were included. Likewise, patients with ongoing or recent antibiotic treatment at risk of getting false negative blood cultures despite showing symptoms of inflammation or sepsis, were included. The retrospective design also forced a non-optimal use of the Shapiro score. Despite these limitations both NLCR and MSS predicted positive blood culture similar to reports in earlier studies [15–18, 20–22, 29] and adds to the body of literature in this topic. The wide inclusion criteria allowed the cohort to mirror an everyday, unbiased influx of patients with suspected infections at the ED. This strengthen the robustness of NLCR and MSS as predictors of positive blood culture in an everyday life setting. Further, the RISE-study did not reach the 100 positive blood cultures as planned, but was closed earlier due to practical reasons and as the number of positive cultures and the species distribution was considered representative and sufficient.

As previous studies have shown BC to be suboptimal as the gold standard in the diagnostics of BSI with/without sepsis [5] the development of new biological markers and etiological diagnostic tests needs clinically relevant and well-defined cohorts of patients with a high probability of BSI. We believe that by applying the predictions presented here on the RISE-cohort we can create such a test-cohort for coming studies. Hence, the group fulfilling Sepsis-3 criteria with NLCR> 12 and MSS ≥ 3p consisted of 48 patients in this study and will together with all the blood culture positive patients in the RISE-cohort be the primary target for future studies on biomarkers and rapid tests for the microbiological diagnosis of BSI.

In this study, we present a well-characterized cohort of patients with suspected blood stream infection. The neutrophil to lymphocyte count ratio (NLCR) and a Modified Shapiro score (MSS) showed capability to predict blood stream infection in the whole cohort as well as in a subgroup of patients fulfilling Sepsis-3 criteria. The observed difference between the MSS and NLCR in the prediction of Gram-positive/Gram-negative BSI is interesting and warrants further studies.

## Supplementary Information

**Additional file 1:** Supplementary Material 1.

**Additional file 2:** Supplementary Material 2.

**Additional file 3:** Supplementary Material 3.

## Data Availability

The datasets used and/or analysed during the current study are available from the corresponding author on reasonable request.

## Abbreviations

BSI: Blood stream infection
BC: Blood culture
PCR: Polymerase chain reaction
NLCR: Neutrophil to lymphocyte count ratio
RISE: Rapid Identification of Sepsis
ED: Emergency department
MSS: Modified Shapiro score
SOFA: Sequential organ failure assessment
RLS: Reaction level scale
IQR: Interquartile range
SD: Standard deviation
AUC: Area under [receiver operating characteristic] curve
CRP: C-reactive protein

## References

[1] Goto M, Al-Hasan MN (2013). Overall burden of bloodstream infection and nosocomial bloodstream infection in North America and Europe. Clin Microbiol Infect.

[2] Laupland KB (2013). Incidence of bloodstream infection: a review of population-based studies. Clin Microbiol Infect.

[3] Singer M, Deutschman CS, Seymour CW, Shankar-Hari M, Annane D, Bauer M, Bellomo R, Bernard GR, Chiche JD, Coopersmith CM, Hotchkiss RS, Levy MM, Marshall JC, Martin GS, Opal SM, Rubenfeld GD, van der Poll T, Vincent JL, Angus DC (2016). The third international consensus definitions for Sepsis and septic shock (Sepsis-3). JAMA..

[4] Kumar A, Roberts D, Wood KE, Light B, Parrillo JE, Sharma S, Suppes R, Feinstein D, Zanotti S, Taiberg L, Gurka D, Kumar A, Cheang M (2006). Duration of hypotension before initiation of effective antimicrobial therapy is the critical determinant of survival in human septic shock. Crit Care Med.

[5] Peters RPH, van Agtmael MA, Danner SA, Savelkoul PHM, Vandenbroucke-Grauls CMJE (2004). New developments in the diagnosis of bloodstream infections. Lancet Infect Dis.

[6] Makristathis A, Harrison N, Ratzinger F, Kussmann M, Selitsch B, Forstner C (2018). Substantial diagnostic impact of blood culture independent molecular methods in bloodstream infections: superior performance of PCR/ESI-MS. Sci Rep.

[7] Ziegler I, Fagerström A, Strålin K, Mölling P (2016). Evaluation of a commercial multiplex PCR assay for detection of pathogen DNA in blood from patients with suspected Sepsis. PLoS One.

[8] Peker N, Couto N, Sinha B, Rossen JW (2018). Diagnosis of bloodstream infections from positive blood cultures and directly from blood samples: recent developments in molecular approaches. Clin Microbiol Infect Off Publ Eur Soc Clin Microbiol Infect Dis.

[9] Jeong S, Park Y, Cho Y, Kim H-S (2012). Diagnostic utilities of procalcitonin and C-reactive protein for the prediction of bacteremia determined by blood culture. Clin Chim Acta Int J Clin Chem.

[10] Hoeboer SH, van der Geest PJ, Nieboer D, Groeneveld ABJ (2015). The diagnostic accuracy of procalcitonin for bacteraemia: a systematic review and meta-analysis. Clin Microbiol Infect Off Publ Eur Soc Clin Microbiol Infect Dis.

[11] Lin C-T, Lu J-J, Chen Y-C, Kok VC, Horng J-T (2017). Diagnostic value of serum procalcitonin, lactate, and high-sensitivity C-reactive protein for predicting bacteremia in adult patients in the emergency department. PeerJ..

[12] Takeshima T, Yamamoto Y, Noguchi Y, Maki N, Gibo K, Tsugihashi Y, Doi A, Fukuma S, Yamazaki S, Kajii E, Fukuhara S (2016). Identifying patients with bacteremia in community-hospital emergency rooms: a retrospective cohort study. PLoS One.

[13] Lee C-C, Wu C-J, Chi C-H, Lee N-Y, Chen P-L, Lee H-C, Chang CM, Ko NY, Ko WC (2012). Prediction of community-onset bacteremia among febrile adults visiting an emergency department: rigor matters. Diagn Microbiol Infect Dis.

[14] Ljungström L, Pernestig A-K, Jacobsson G, Andersson R, Usener B, Tilevik D. Diagnostic accuracy of procalcitonin, neutrophil-lymphocyte count ratio, C-reactive protein, and lactate in patients with suspected bacterial sepsis. PLoS One. 2017;12(7):e0181704. 10.1371/journal.pone.0181704 PMC5519182 28727802

[15] Shapiro NI, Wolfe RE, Wright SB, Moore R, Bates DW (2008). Who needs a blood culture? A prospectively derived and validated prediction rule. J Emerg Med.

[16] Jessen M, Mackenhauer J, Hvass AM, Ellermann-Eriksen S, Skibsted S, Kirkegaard H, Schønheyder HC, Shapiro NI, CONSIDER Sepsis Network (2016). Prediction of bacteremia in the emergency department: an external validation of a clinical decision rule. Eur J Emerg Med.

[17] Hodgson LE, Dragolea N, Venn R, Dimitrov BD, Forni LG (2016). An external validation study of a clinical prediction rule for medical patients with suspected bacteraemia. Emerg Med J.

[18] Laukemann S, Kasper N, Kulkarni P, Steiner D, Rast AC, Kutz A, Felder S, Haubitz S, Faessler L, Huber A, Fux CA, Mueller B, Schuetz P (2015). Can we reduce negative blood cultures with clinical scores and blood markers? Results from an observational cohort study. Medicine (Baltimore).

[19] Zahorec R (2001). Ratio of neutrophil to lymphocyte counts--rapid and simple parameter of systemic inflammation and stress in critically ill. Bratisl Lek Listy.

[20] Lowsby R, Gomes C, Jarman I, Lisboa P, Nee PA, Vardhan M, Eckersley T, Saleh R, Mills H (2015). Neutrophil to lymphocyte count ratio as an early indicator of blood stream infection in the emergency department. Emerg Med J EMJ.

[21] de Jager CP, van Wijk PT, Mathoera RB, de Jongh-Leuvenink J, van der Poll T, Wever PC (2010). Lymphocytopenia and neutrophil-lymphocyte count ratio predict bacteremia better than conventional infection markers in an emergency care unit. Crit Care.

[22] Loonen AJM, de Jager CPC, Tosserams J, Kusters R, Hilbink M, Wever PC, van den Brule AJC (2014). Biomarkers and molecular analysis to improve bloodstream infection diagnostics in an emergency care unit. PLoS One.

[23] Universitetssjukhuset Örebro - About Us: Pettersson, A. [cited 2019 04-10]. Available from: https://www.regionorebrolan.se/en/Orebro.University-hospital/.

[24] Charlson M, Pompei P, Ales KL, Mackenzie C (1987). A new method of classifying prognostic comorbidity in longitudinal studies: development and validation. J Chronic Dis.

[25] Walther SM, Jonasson U, Gill H (2003). Comparison of the Glasgow coma scale and the reaction level scale for assessment of cerebral responsiveness in the critically ill. Intensive Care Med.

[26] DeLong ER, DeLong DM, Clarke-Pearson DL (1988). Comparing the areas under two or more correlated receiver operating characteristic curves: a nonparametric approach. Biometrics..

[27] Kim S, Lee W (2017). Does McNemar’s test compare the sensitivities and specificities of two diagnostic tests?. Stat Methods Med Res.

[28] Park HK, Kim WY, Kim MC, Jung W, Ko BS (2017). Quick sequential organ failure assessment compared to systemic inflammatory response syndrome for predicting sepsis in emergency department. J Crit Care.

[29] Marik PE, Stephenson E (2020). The ability of Procalcitonin, lactate, white blood cell count and neutrophil-lymphocyte count ratio to predict blood stream infection. Analysis of a large database. J Crit Care.

[30] Rosenqvist M, Bengtsson-Toni M, Tham J, Lanbeck P, Melander O, Åkesson P. Improved Outcomes After Regional Implementation of Sepsis Alert: A Novel Triage Model. Crit Care Med. 2020;48(4):484–90. 10.1097/CCM.0000000000004179 32205594

[31] Liu Z, Meng Z, Li Y, Zhao J, Wu S, Gou S, Wu H (2019). Prognostic accuracy of the serum lactate level, the SOFA score and the qSOFA score for mortality among adults with Sepsis. Scand J Trauma Resusc Emerg Med.

[32] Drees M, Kanapathippillai N, Zubrow MT (2012). Bandemia with Normal White Blood Cell Counts Associated with Infection. Am J Med.

[33] Turak O, Özcan F, İşleyen A, Başar FN, Gül M, Yilmaz S, Sökmen E, Yüzgeçer H, Lafçi G, Topaloğlu S, Aydoğdu S (2013). Usefulness of neutrophil-to-lymphocyte ratio to predict in-hospital outcomes in infective endocarditis. Can J Cardiol.

[34] Poutsiaka DD, Porto MC, Perry WA, Hudcova J, Tybor DJ, Hadley S, et al. Prospective observational study comparing Sepsis-2 and Sepsis-3 definitions in predicting mortality in critically ill patients. Open Forum Infect Dis. 2019;6(7). 10.1093/ofid/ofz271 PMC6602380 31281865

