# Supplementary material for: Early prediction of blood stream infection in a prospectively collected cohort
Source: BMC Infect Dis. 2021 Apr 2;21:316. doi: 10.1186/s12879-021-05990-3 (PMC8017733; doi:10.1186/s12879-021-05990-3)
Supplement: Supplementary file 1 — Additional file 1: Supplementary Material 1. [file 12879_2021_5990_MOESM1_ESM.docx]

**Supplementary Material 1**

Structured list of all variables retrospectively extracted from the RISE cohort.

1. Demographics
   1. Gender (male, female)
   2. Age (years)
2. Comorbidities
   1. Diabetes (Y/N)
      1. With or without diagnosed secondary complications
   2. Heart failure (Y/N)
   3. Chronic lung disease (e.g. asthma, chronic obstructive pulmonary disease etc.) (Y/N)
   4. Kidney failure (Y/N)
   5. Liver failure (Y/N)
   6. Malignancy (solid, non-solid) (Y/N)
   7. Hemi-/paraplegia (Y/N)
   8. Ischemic heart disease (Y/N)
   9. Myocardial infarction in medical history (Y/N)
   10. Peptic ulcer disease (Y/N)
   11. Dementia (Y/N)
   12. Rheumatic disease (e.g systemic lupus erythematosus, rheumatoid arthritis, polymyalgia rheumatic etc.) (Y/N)
   13. Cerebrovascular disease (stroke or transient ischemic attack (TIA) in medical history) (Y/N)
   14. HIV/AIDS (Y/N)
   15. Hypogammaglobulinemia (Y/N)
   16. Splenectomy in medical history (Y/N)
3. Recent contact with hospital care at time of inclusion
   1. Surgery or trauma < 3 days prior to inclusion (Y/N)
   2. Inpatient treatment < 48 h prior to inclusion (Y/N)
4. Other important medical circumstances
   1. Ongoing medical treatment
      1. Anticoagulant treatment (Vitamin K antagonists, Novel Oral Anticoagulants, Acetyl salicylic acid) (Y/N)
      2. Corticosteroid treatment (> 20 mg prednisone daily) (Y/N)
      3. Other immunosuppressant drugs (prescribed drug, dose)
      4. Antibiotics (current treatment and/or treatment within the last 14 days). (Y/N, prescribed drug)
   2. Indwelling dialysis catheter (Y/N)
   3. Indwelling subcutan vein catheter (Y/N)
5. Debut of systemic symtoms prior to inclusion
   1. Debut of systemic symtoms (date)
   2. Arrival at the emergency department – (date, time)
   3. Initial assessment of ED nurse – (date, time)
6. Symtoms
   1. Chills (Y/N)
   2. Cough (Y/N)
   3. Dyspnea (Y/N)
   4. Nausea/vomiting (Y/N)
   5. Diarrhea (Y/N)
   6. Skin rash (Y/N)
   7. Abscess (Y/N)
   8. Recent onset confusion (Y/N)
   9. Pain – thorax, stomach, head, joints, extremities (Y/N, specified location)
   10. Urge to urinate or painful and burning sensation when urinating (Y/N)
7. Vital parameters at arrival and any deterioration during stay at the ED
   1. Alertness (Reaction Level Scale)
   2. Pulse frequency (Beats per minute)
   3. Blood pressure (mmHg)
   4. Respiratory rate, oxygen saturation (breaths/minute, SaO2%)
   5. Body temperature (°C)
   6. Modified early warning scale (MEWS)
8. Chemical laboratory tests
   1. White blood cells, total and differential count – (cells/mm^3^)
   2. Thrombocyte particle count (particles/mm^3^)
   3. C-reactive protein (mg/L)
   4. Creatinine/estimated GFR (µmol/L; mL/min/1,73m^2^)
   5. Prothrombin Complex concentrate (international normalized ratio)
   6. Bilirubin (µmol/L)
   7. Lactate (mmol/L)
   8. Base excess (mmol/L)
   9. Procalcitonin (µg/L)
9. Primary suspected infection site (> one option possible/patient)
   1. Abscess (Y/N)
   2. Endocarditis (Y/N)
   3. Meningitis/CNS-infection (Y/N)
   4. Soft tissue infection other than abscess (Y/N)
   5. Osteomyelitis (Y/N)
   6. Prosthetic joint infection (Y/N)
   7. Urinary tract infection (Y/N)
   8. Pneumonia (Y/N)
10. Radiological investigations
    1. Chest x-ray performed within 24 h (findings)
    2. Other radiological investigations within 24h (findings)
11. Patient care
    1. Time to initiation of treatment
       1. Fluid resuscitation (minutes)
       2. Intravenous antibiotic treatment (minutes)
    2. In-patient care (days)
    3. Admission to the Intensive Care Unit within 24 hours (Y/N)
       1. Use of vasopressor drugs (Y/N)
    4. Antibiotic treatment after inclusion
       1. Prescribed drug
       2. Treatment initiation/cancelling date
       3. Any following changes in antibiotic regimen.
12. Mikrobiological investigations
    1. Blood culture
       1. Time to disposal of blood culture bottles
       2. Blood culture result/detected pathogen.
    2. Other cultures
       1. Urin, wound, airway, faecal cultures
       2. Culture results/detected pathogen.
