# Supplementary material for: Early prediction of blood stream infection in a prospectively collected cohort
Source: BMC Infect Dis. 2021 Apr 2;21:316. doi: 10.1186/s12879-021-05990-3 (PMC8017733; doi:10.1186/s12879-021-05990-3)
Supplement: Supplementary file 2 — Additional file 2: Supplementary Material 2. [file 12879_2021_5990_MOESM2_ESM.docx]

| Sensitivity, specificity, positive (PPV) and negative (NPV) predictive values and positive (LR+) and negative (LR-) likelihood ratios for predicting positive blood culture using Neutrophil to lymphocyte count ratio (NLCR) and Modified Shapiro score (MSS). NLCR > 19.25 and MSS ≥ 4 p presented here were optimal cut-offs for prediction of positive blood culture among patients fulfilling Sepsis-3 criteria based on receiver operator characteristics calculations in this study. Values presented in the whole cohort and among patients fulfilling Sepsis-3 criteria. 95% confidence interval presented within brackets. | | | | | | | | | | | | |
| --- | --- | --- | --- | --- | --- | --- | --- | --- | --- | --- | --- | --- |
|  | Whole cohort n=484 (positive blood cultures n=84 (17%)) | | | | | | Patients fulfilling Sepsis-3 criteria n=155 (positive blood cultures n=32 (21%)) | | | | | |
|  | Sensitivity (%) | Specificity (%) | PPV (%) | NPV (%) | LR+ | LR- | Sensitivity% | Specificity% | PPV (%) | NPV (%) | LR+ | LR- |
| **NLCR > 19.25** | 41 (31-53) | 87 (83-90) | 41 (32-50) | 88 (85-89) | 3.2 (2.2-4.6) | 0.7 (0.6-0.8) | 52 (33-70) | 76 (68-83) | 36 (26-47) | 86 (81-90) | 2.2 (1.4-3.5) | 0.6 (0.4-0.9) |
| **MSS ≥ 4 p** | 50 (39-61) | 81 (77-85) | 35 (29-42) | 89 (86-91) | 2.6 (1.9-3.5) | 0.6 (0.5-0.8) | 66 (47-81) | 71 (62-79) | 37 (29-46) | 89 (83-93) | 2.2 (1.5-3.3) | 0.5 (0.3-0.8) |
|  |  |  |  |  |  |  |  |  |  |  |  |  |

**Supplementary Material 2**
