# Supplementary material for: Early prediction of blood stream infection in a prospectively collected cohort
Source: BMC Infect Dis. 2021 Apr 2;21:316. doi: 10.1186/s12879-021-05990-3 (PMC8017733; doi:10.1186/s12879-021-05990-3)
Supplement: Supplementary file 3 — Additional file 3: Supplementary Material 3. [file 12879_2021_5990_MOESM3_ESM.docx]

| Sensitivity, specificity, positive (PPV) and negative (NPV) predictive values and positive (LR+) and negative (LR-) likelihood ratios for predicting positive blood culture using different Neutrophil to lymphocyte count ratio (NLCR) and Modified Shapiro score (MSS) cut-off values. Values presented among patients not fulfilling Sepsis-3 criteria. 95% confidence presented within brackets. | | | | | | |
| --- | --- | --- | --- | --- | --- | --- |
|  | Patients not fulfilling Sepsis-3 criteria n=329 (positive blood cultures n=52 (16%)) | | | | | |
|  | Sensitivity (%) | Specificity (%) | PPV (%) | NPV (%) | LR+ | LR- |
| NLCR > 10 | 74 (60-85) | 67 (61-73) | 30 (25-36) | 93 (89-95) | 2.3 (1.8-2.9) | 0.4 (0.3-0.6) |
| NLCR > 12 | 69 (54-81) | 73 (68-79) | 33 (28-40) | 92 (89-95) | 2.6 (2.0-3.4) | 0.4 (0.3-0.6) |
| NLCR > 19.25 | 35 (22-50) | 92 (88-95) | 46 (33-60) | 88 (86-90) | 4.4 (2.6-7.7) | 0.7 (0.6-0.9) |
| MSS ≥ 2 p | 81 (67-90) | 44 (38-50) | 21 (19-24) | 92 (87-96) | 1.4 (1.2-1.7) | 0.4 (0.2-0.8) |
| MSS ≥ 3 p | 63 (49-76) | 73 (67-78) | 29 (24-36) | 91 (87-93) | 2.2 (1.7-2.9) | 0.5 (0.4-0.8) |
| MSS ≥ 4 p | 38 (25-53) | 84 (79-88) | 34 (25-44) | 88 (86-91) | 2.7 (1.8-4.2) | 0.7 (0.6-0.9) |
| NLCR > 12 and MSS ≥ 3 | 45 (31-60) | 89 (85-93) | 45 (34-57) | 89 (87-92) | 4.3 (2.7-6.8) | 0.6 (0.5-0.8) |

**Supplementary Material 3**
